# Supersulfide donors and their therapeutic targets in inflammatory diseases

**DOI:** 10.3389/fimmu.2025.1581385

**Published:** 2025-04-16

**Authors:** Tianli Zhang, Yuexuan Pan, Tomohiro Sawa, Takaaki Akaike, Tetsuro Matsunaga

**Affiliations:** ^1^ Center for Integrated Control, Epidemiology and Molecular Pathophysiology of Infectious Diseases, Akita University, Akita, Japan; ^2^ Department of Redox Molecular Medicine, Tohoku University Graduate School of Medicine, Sendai, Japan; ^3^ Department of Microbiology, Graduate School of Medical Sciences, Kumamoto University, Kumamoto, Japan; ^4^ Shimadzu × Tohoku University Supersulfides Life Science Co-creation Research Center, Sendai, Japan

**Keywords:** supersulfides, hydropersulfides, hydropolysulfides, persulfides, polysulfides, supersulfide donors, inflammation, inflammatory responses

## Abstract

Inflammation is one defense mechanism of the body that has multiple origins, ranging from physical agents to infectious agents including viruses and bacteria. The resolution of inflammation has emerged as a critical endogenous process that protects host tissues from prolonged or excessive inflammation, which can become chronic. Failure of the inflammation resolution is a key pathological mechanism that drives the progression of numerous inflammatory diseases. Owing to the various side effects of currently available drugs to control inflammation, novel therapeutic agents that can prevent or suppress inflammation are needed. Supersulfides are highly reactive and biologically potent molecules that function as antioxidants, redox regulators, and modulators of cell signaling. The catenation state of individual sulfur atoms endows supersulfides with unique biological activities. Great strides have recently been made in achieving a molecular understanding of these sulfur species, which participate in various physiological and pathological pathways. This review mainly focuses on the anti-inflammatory effects of supersulfides. The review starts with an overview of supersulfide biology and highlights the roles of supersulfides in both immune and inflammatory responses. The various donors used to generate supersulfides are assessed as research tools and potential therapeutic agents. Deeper understanding of the molecular and cellular bases of supersulfide-driven biology can help guide the development of innovative therapeutic strategies to prevent and treat diseases associated with various immune and inflammatory responses.

## 1 Introduction

Inflammation, which was first documented by Cornelius Celsus in the 1st century AD and today remains one of the most pressing unsolved medical issues, affects millions of people worldwide (1). Despite inflammation is characterized by five symptoms, including redness, swelling, heat, pain, and loss of tissue function, the definition and mechanism of inflammation are extraordinarily complex (1). Inflammation can be classified on the basis of its origin—arising from either external agents or endogenous abnormal responses—and its duration, that is, acute or chronic (2). Acute inflammation is typically a protective innate immune response triggered by infection or injury (3), whereas chronic inflammation often accompanies pathological states, such as aging, without any infection or injury (4). Current understanding posits that inflammation is initiated by sentinel cells that detect tissue stress and deviations from homeostasis. The molecules involved in this process act to restore normal functions (5). Indeed, proper inflammatory responses activate innate immune defenses and coordinate adaptive immunity against specific pathogens. To achieve these defensive responses, the host immune system employs multiple regulatory mechanisms that modulate the initiation, progression, and resolution of inflammation (6). In most cases, the acute inflammation resolves once the harmful stimulus is eliminated and damaged tissue is repaired. However, this tightly regulated process can be disrupted, leading to persistent unresolved inflammation. Prolonged inflammation not only harms tissues but also contributes to the development and progression of chronic diseases associated with disrupted homeostasis (6). Recent studies have highlighted unresolved inflammation as a causal factor in numerous human diseases, ranging from psychiatric disorders to infectious disorders, emphasizing the urgent need to develop effective strategies to control inflammation (7).

**Figure 1:**
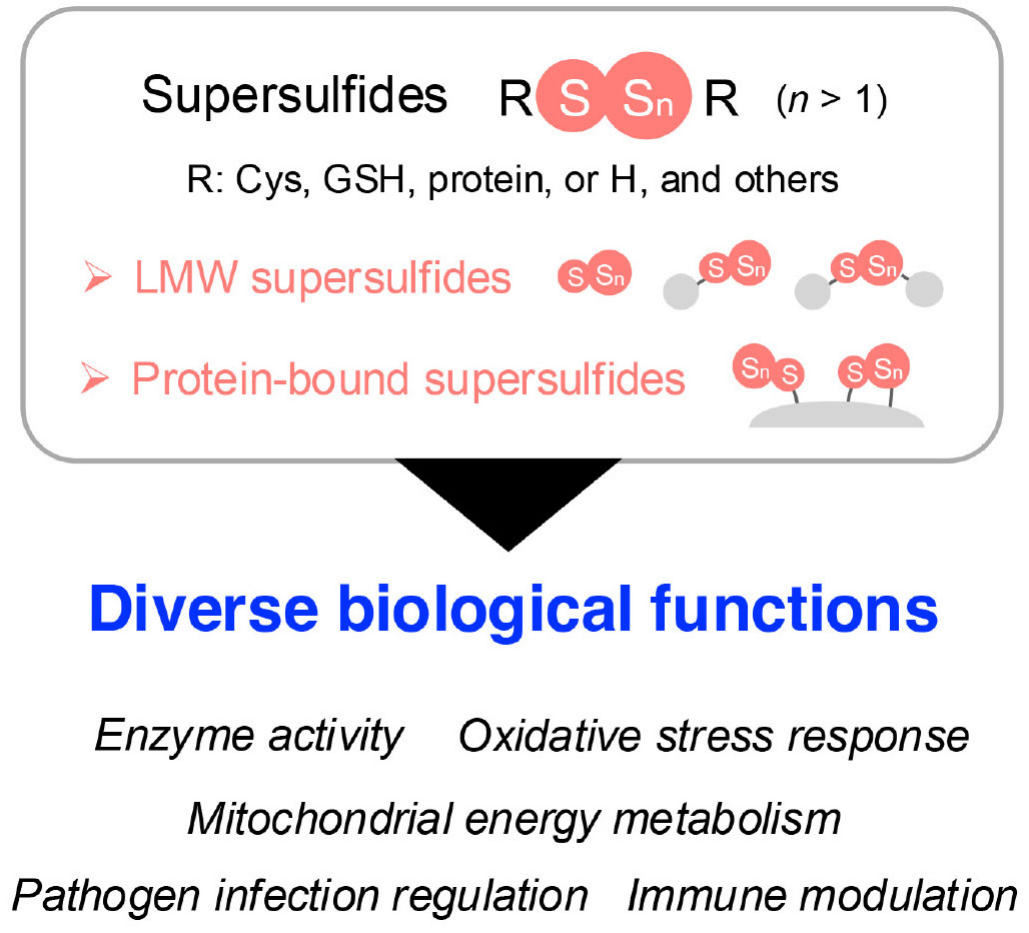
Overview of supersulfides and their biological functions. Supersulfides are classified into LMW supersulfides and protein-bound supersulfides, both of which play vital roles in cellular processes. These processes include regulating enzyme activity, managing oxidative stress, participating in energy metabolism, and modulating immune responses. Cys, cysteine; GSH, glutathione; H, hydrogen; LMW, low-molecular-weight.

Sulfur is an essential element for all living organisms because of its incorporation into amino acids, proteins, and various biomolecules (8). As the third most abundant mineral in the human body, sulfur and its signaling play pivotal roles in a wide range of physiological and pathological processes, such as cell signaling, energy production, free radical detoxification, and protein structural integrity (9). Early research in sulfur signaling primarily focused on hydrogen sulfide (H_2_S), which is often referred to as the third gasotransmitter, although its classification remains debated (10). However, advancements in mass spectrometry (MS)-based quantitative metabolomic techniques have revealed the widespread presence of diverse sulfur-containing species in biological systems, each exhibiting distinct functions as a result of their unique chemical properties (11–13). Among these species, supersulfides—including hydropersulfides, hydropolysulfides (RSS_n_H, *n* > 1), polysulfides (RSS_n_R, *n* > 1), and inorganic persulfides and polysulfides—have recently been identified as endogenous molecules characterized by catenated sulfur chains in their structures (14). The catenation of sulfur atoms endows supersulfides with unique reactivity as it positions them as critical regulators in various biological processes, such as enzyme activity, oxidative stress response, energy metabolism, pathogen infection, and immune modulation (15–17) (**Figure 1**).

This review aims to describe the emerging therapeutic potential of supersulfides in inflammation-associated diseases. It begins with a summary of current biological insights into supersulfides. This summary is followed by an overview of pattern recognition receptor (PRR)-mediated inflammatory responses, the pathogenesis of several representative inflammation-associated diseases, and existing anti-inflammatory therapies. Additionally, the anti-inflammatory effects of both natural and synthetic supersulfide donors are discussed, alongside recent evidence supporting the involvement of supersulfides in inflammation regulation. To promote the development of this promising class of anti-inflammatory compounds into licensed therapeutics, several critical directions for future research are proposed. A deeper understanding of the molecular and cellular mechanisms underlying supersulfide biology will pave the way for development of innovative therapeutic strategies to prevent and treat diseases driven by dysregulated immune and inflammatory responses.

## 2 Endogenous occurrence of supersulfides across organisms

Advances in MS-based metabolomics have led to the demonstration of the widespread presence of low-molecular-weight (LMW) supersulfides in a range of organisms, including yeast, bacteria, mammals, and humans. Typical LMW supersulfides include cysteine (CysSH)-based cysteine persulfide/polysulfide (CysSSH/CysSS_n_H, *n* > 1), glutathione persulfide/polysulfide (GSSH/GSS_n_H, *n* > 1), and cysteine trisulfide, as well as glutathione trisulfide (GSSSG) (18). Inorganic supersulfides, such as hydrogen disulfide (H_2_S_2_) and polysulfides (H_2_S_n_, *n* > 1), have also been reported as possibly present in biological systems (18). In addition to LMW supersulfide forms, protein-bound supersulfides, in which supersulfides are linked through sulfur catenation of thiol in CysSH residues, have been identified (19, 20) (**Figure 1**).

**Figure 2:**
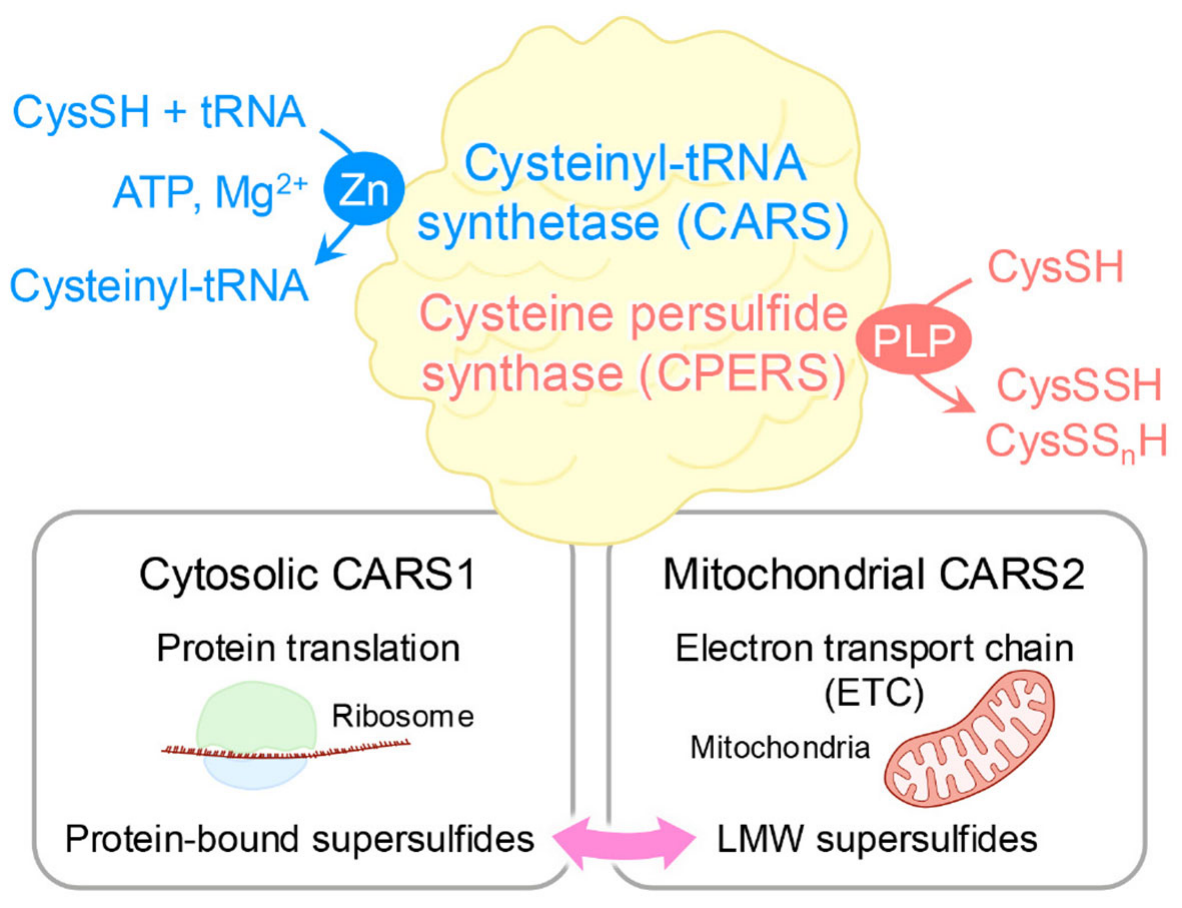
Dual enzymatic functions of CARS. As a cysteinyl-tRNA synthetase, CARS performs dual enzymatic functions as it catalyzes the formation of tRNA-bound CysSSH adducts, which enables the incorporation of CysSSH into proteins and promotes the generation of protein-bound supersulfides. In addition, through a pyridoxal phosphate (PLP)-dependent reaction, CARS synthesizes CysSSH by utilizing a second cysteine molecule as the sulfur donor, independent of ATP and tRNA. This activity contributes to sulfur-oxygen hybrid respiration within mitochondria.

These supersulfides, regardless of their forms, can be generated via enzymatic or chemical processes. At first, cystathionine β-synthase and cystathionine γ-lyase were believed to be the key enzymes responsible for supersulfide production via the transsulfuration pathway (21). These enzymes catalyze the conversion of CysSH to CysSSH, which can undergo disproportionation to form oxidized species, such as cysteine trisulfide, along with the release of H_2_S. H_2_S can then participate in more complex supersulfide generation through thiol exchange reactions. Similarly, glutathione (GSH) reacts with CysSSH to produce GSSH, which undergoes analogous transformations. GSSH can also be produced by means of the glutathione reductase-mediated reduction of oxidized glutathione polysulfides (21). Moreover, 3-mercaptopyruvate sulfurtransferase generates both LMW and protein-bound supersulfides via the transsulfuration pathway (22, 23). As an interesting result, a study using triple-knockout mice lacking cystathionine β-synthase, cystathionine γ-lyase, and 3-mercaptopyruvate sulfurtransferase revealed that supersulfides were still produced, suggesting the existence of compensatory or alternative synthetic pathways for supersulfide generation (24). In support of this idea, one such alternative mechanism involves cysteinyl-tRNA synthetase (CARS), which is an enzyme that is well-known for catalyzing the synthesis of cysteinyl-tRNA (25). Eukaryotic cells express two CARS isoforms: CARS1, localized in the cytosol, and CARS2, localized in mitochondria (26). Recent work by Akaike and colleagues identified CARS as a novel cysteine persulfide synthase (CPERS), which can directly produce supersulfides by using CysSH as a substrate. With the use of recombinant CARS proteins from mice (CARS1), humans (CARS2), and *Escherichia coli* (EcCARS), the study demonstrated conserved CPERS activity across species (15). Although deletion of CARS2 in mice is embryonic lethal, mice with a heterozygous knockout of CARS2 showed approximately 50% lower levels of supersulfides compared with wild-type mice, indicating a predominant role of mitochondrial CARS2 in supersulfide production (15) (**Figure 2**).

It is worth noting that supersulfides, on appearance, constitute only a small percentage of their corresponding parental molecules, such as CysSH and GSH, and share similar chemical structures, with the manifestation of some overlapping physiological effects in mammalian cells (15, 21, 27). However, supersulfides often demonstrate greater activities than do their parental counterparts. For example, GSSH have a higher reducing ability to reduce hydrogen peroxide, whereas GSH is ineffective in this regard (21). The enhanced reactivity of GSSH can be attributed to a lower p*Ka* value. That is, the p*Ka* of GSSH is 6.9, which is two orders of magnitude lower than that of GSH, which is 8.9 (28). In other words, this lower p*Ka* implies that supersulfides have a greater tendency to deprotonate and form anionic species under physiological conditions, thereby increasing their reactivity with electrophilic molecules. This feature likely accounts for the higher reactivity of GSSH compared with GSH. Supporting this, H_2_S_n_ compounds have been found to induce calcium mobilization by activating transient receptor potential A1 channels and show a potency that is 320 times greater than that of parental H_2_S (27).

As mentioned above, protein-bound supersulfides were identified in various proteins, where they modulate protein functions by regulating the CysSH residues within these proteins (19, 20, 29–34). Protein persulfides were traditionally thought to be formed via thiol modifications mediated by sulfur-containing molecules such as H_2_S (35). However, a controversial study reported that H_2_S_n_ species modify glyceraldehyde 3-phosphate dehydrogenase (GAPDH) activity through CysSH persulfidation, whereas H_2_S itself cannot do so (36). Such results are understandable given that the oxidation state of H_2_S does not permit it to directly persufidate a thiol group unless it undergoes prior oxidation. Instead of H_2_S, GSSH can efficiently donate sulfur atoms to acceptor protein thiols, which would facilitate the formation of protein-bound supersulfides (21). In general, CARSs are enzymes that catalyze cysteinyl-tRNA production by means of a two-step mechanism. CysSH is first activated in the presence of ATP to form an enzyme-bound cysteinyl adenylate, followed by the transfer of the activated CysSH to the 3’-terminus of cysteinyl-tRNA (25). More importantly, CysSSH bound to cysteinyl-tRNA was discovered in *in vitro* reactions involving CysSH, CARS, cysteinyl-tRNA, and ATP (15). Moreover, an analysis of nascent polypeptides synthesized by *E. coli* ribosomes revealed the extensive formation of peptide persulfides, suggesting that protein supersulfidation occurs during protein synthesis (15) (**Figure 2**). These findings suggest that supersulfides play a crucial role in regulating the function of proteins across a wide variety of biological contexts.

## 3 A spectrum of inflammatory responses and the key pathways involved

**Figure 3:**
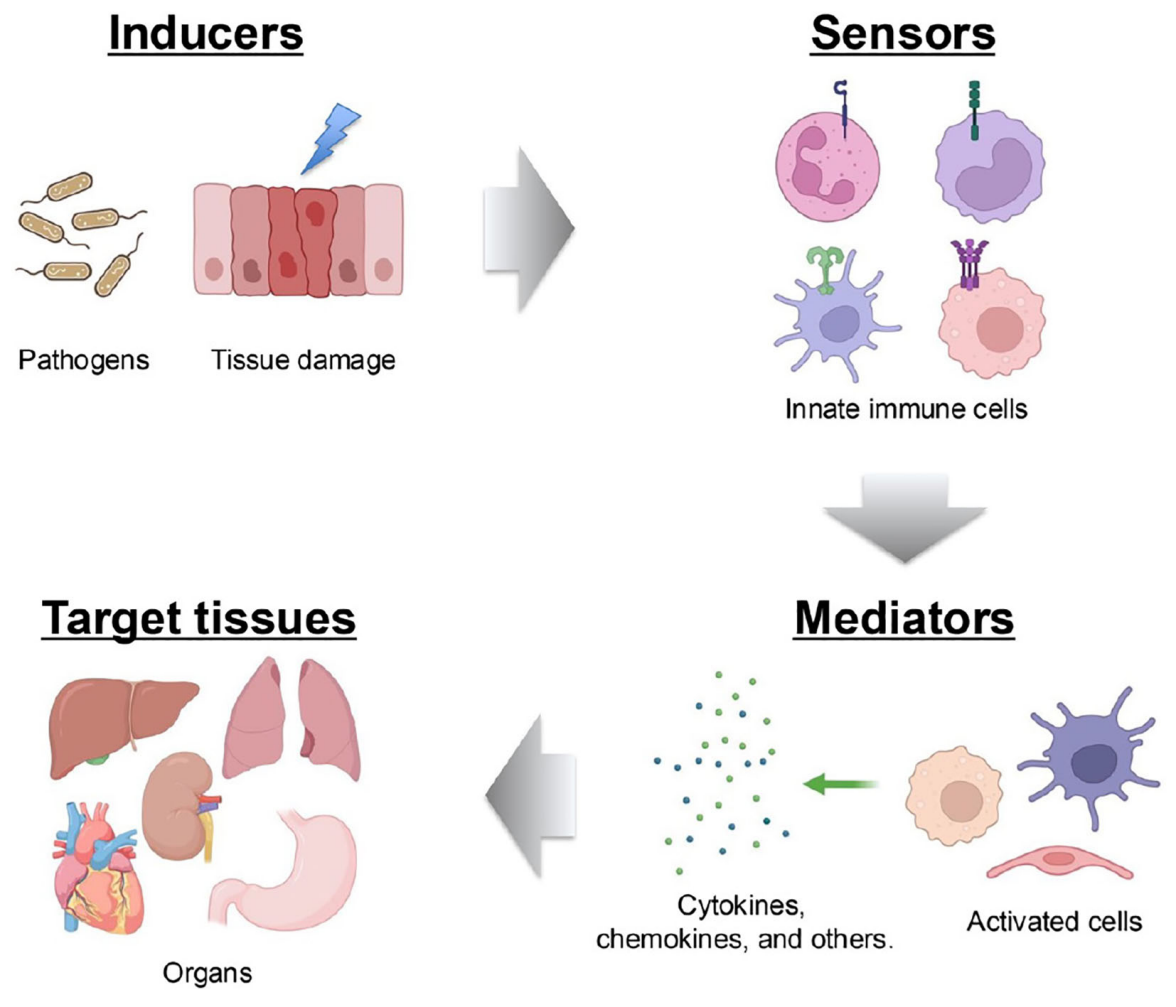
Components of the inflammatory process. A typical inflammatory pathway consists of inducers, sensors, mediators and effectors. Inflammation is initiated by infections and tissue damage, which are recognized by PRRs. This recognition triggers the secretion of immune mediators, such as cytokines, which subsequently affect target tissues.

An inflammatory response typically comprises four key components: *inducers* that initiate signaling cascades, *sensors* that link inducers to signaling pathways, *mediators* that are produced as a result of signaling, and *targets* that are affected by these mediators (37). Each component exists in diverse forms, and their combinations define distinct inflammatory pathways. At its core, the innate immune system—that is, cells such as monocytes, macrophages, dendritic cells, and neutrophiles—plays a pivotal role in mounting inflammatory responses triggered by microbial or non-microbial stimuli (38). These immune cells recognize conserved molecular patterns on pathogens, which are known as pathogen-associated molecular patterns (PAMPs), by using germline-encoded PRRs. This recognition occurs during infections caused by bacteria, viruses, or fungi, enabling a rapid inflammatory response before the pathogen inflicts significant harm (39). In addition to PAMPs, the host generates damage-associated molecular patterns (DAMPs) in response to tissue injury, cell death, and other stressors (40). PRRs also detect these host-derived molecules to initiate inflammation (41). Four major PRR families have now been identified: Toll-like receptors (TLRs) and C-type lectin receptors, which are transmembrane proteins, and cytoplasmic retinoic acid-inducible gene (RIG-I)-like receptors (RLRs) and nucleotide oligomerization domain-like receptors (NLRs) (41). Activation of PRRs by their respective ligands triggers the transcription of genes involved in inflammatory responses. Although the expression patterns of these genes vary, they predominantly encode pro-inflammatory cytokines, type I interferons (IFN-I) (e.g., IFN-α and IFN-β), chemokines, and proteins that modulate PRR-mediated signaling, which collectively shape the inflammatory process (42) (**Figure 3**).

### 3.1 TLR-nuclear factor kappa B signaling pathway

As one of the earliest identified PRR families, TLRs comprise 10 members in humans (TLR1-10) and 12 in mice (TLR1-9, 11-13) (43, 44). TLRs are divided into two groups on the basis of their localization: cell-surface TLRs, such as TLR1, TLR2, TLR4, TLR5, TLR6, and TLR10, which primarily recognize microbial membrane components like lipids and lipoproteins, and intracellular TLRs. For instance, TLR2 recognizes zymosan, while TLR4 detects lipopolysaccharide (LPS) (45). In contrast, intracellular TLRs mainly identify microbial nucleic acids (46, 47). For example, TLR3 senses viral double-stranded RNA (dsRNA) and self-RNAs (45). Although different TLRs recognize distinct ligands, all TLRs share a conserved cytoplasmic domain named Toll/interleukin (IL)-1 receptor (TIR) domain, which mediates downstream signaling by recruiting specific adaptor proteins, including TIR domain-containing adaptor molecules to receptors. These receptors include myeloid differentiation primary response 88 (MyD88), TIR domain-containing adaptor-inducing interferon-β (TRIF), TIR domain-containing adaptor protein (TIRAP), and TRIF-related adaptor molecule (TRAM) (48). TLR signaling can thus be placed into one of two pathways: MyD88-dependent pathways and TRIF-dependent pathways. After binding to ligands, TLRs dimerize, allowing their TIR domains to associate with adaptor proteins. MyD88-dependent signaling, either directly or via TIRAP, leads to the assembly of IL-1 receptor-associated kinases (IRAKs) and tumor necrosis factor (TNF)-associated factor 6 (TRAF6), thereby culminating in the phosphorylation of inhibitor κ B kinases (IKKs). IKKs phosphorylate nuclear factor kappa B (NF-κB)-inhibitory protein (IκBα), making it for degradation and thereby releasing NF-κB; NF-κB translocates to the nucleus to drive expression of pro-inflammatory genes such as TNF-α (49).

**Figure 4:**
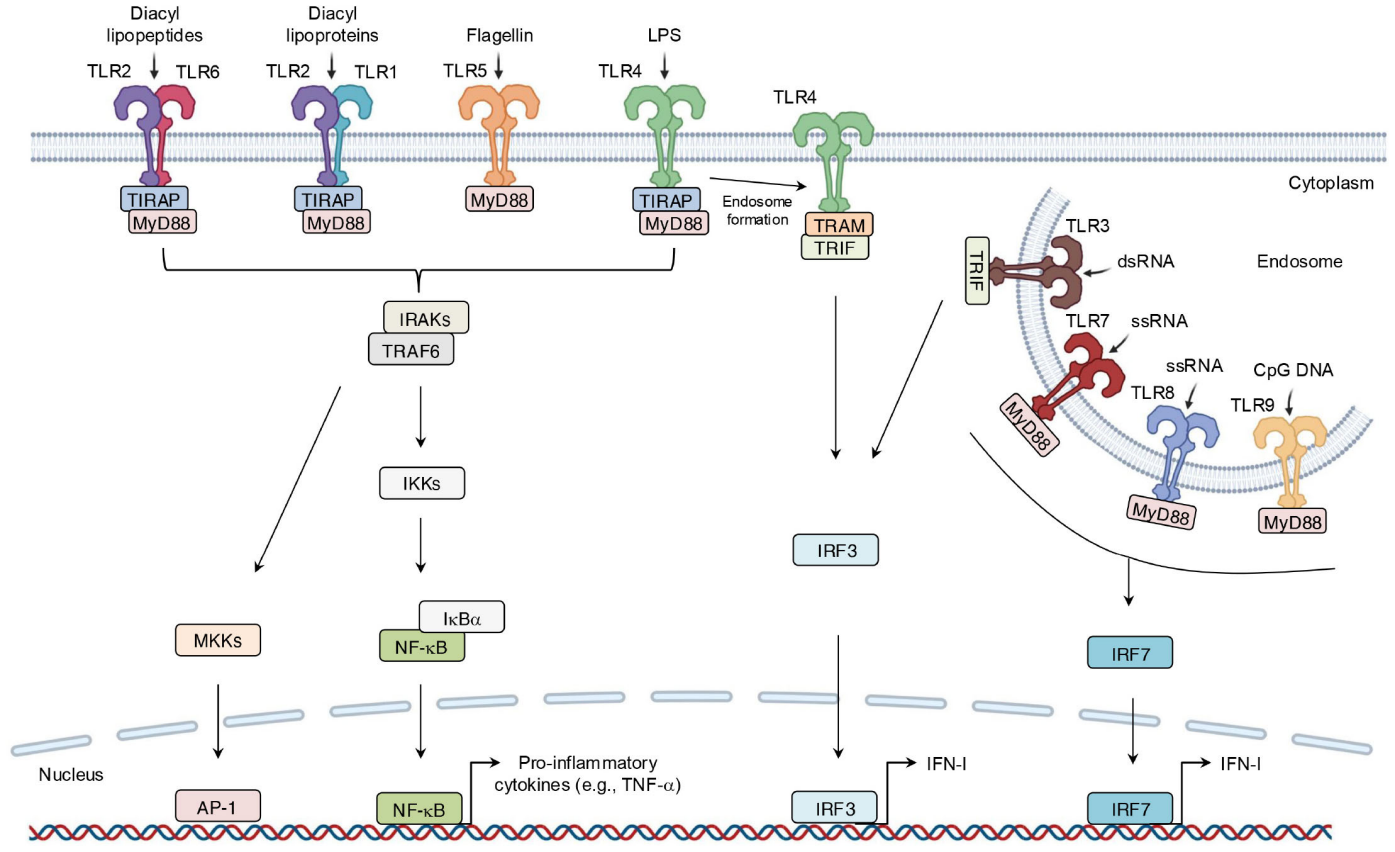
Binding of ligand-TLRs and their signaling transduction. TLRs recognize their specific ligands, typically dimerize on activation, and recruit adaptor molecules containing the same TIR domain to transmit signals. This process ultimately leads to the production of inflammatory mediators.

**Figure 5:**
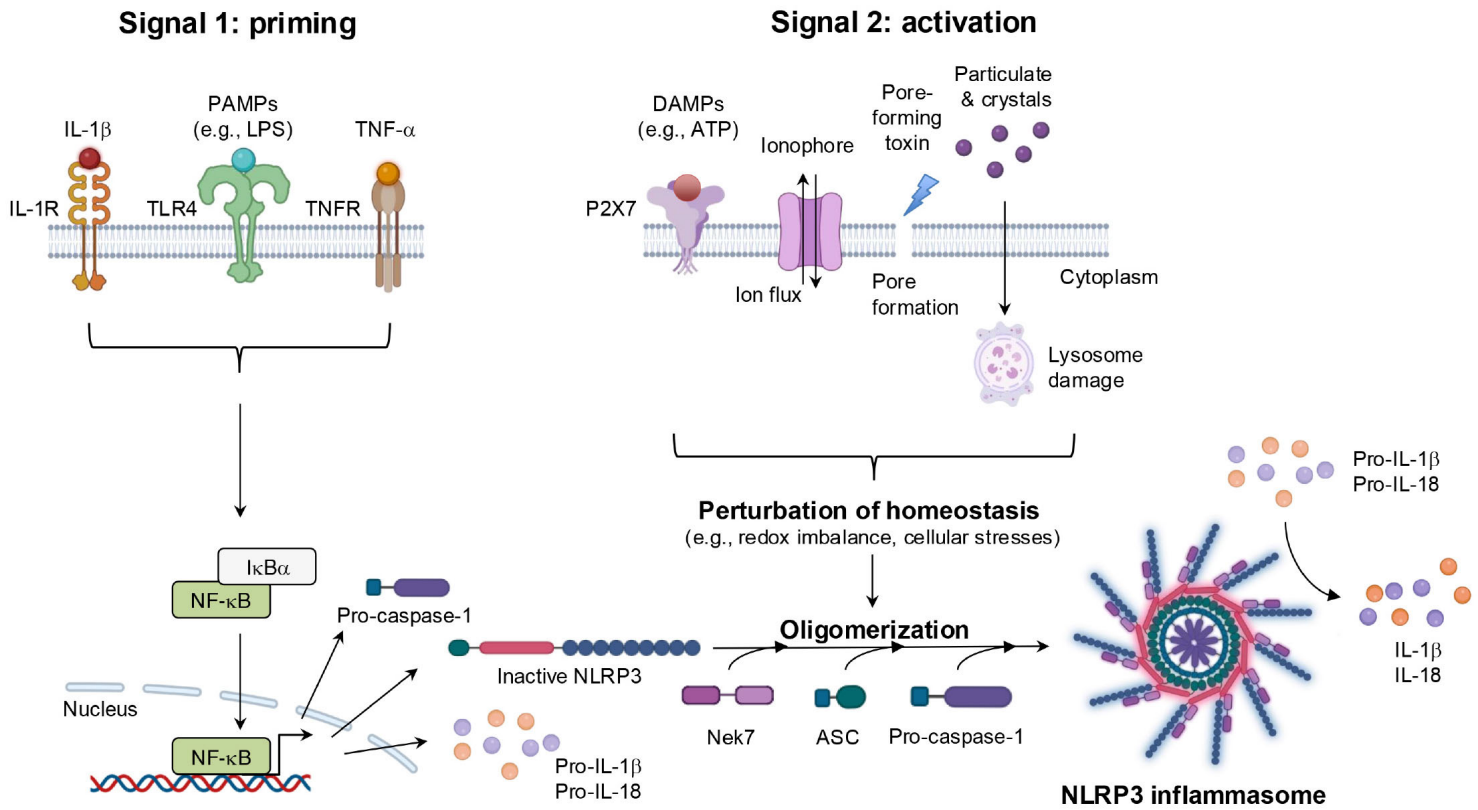
Inhibitory effects of supersulfides on the TLR signaling pathway. Administration of NAC-S2 significantly improved the survival rate of mice subjected to lethal endotoxin shock. Mechanistically, TLR ligands, including zymosan A, polyinosinic-polycytidylic acid sodium salt (poly I:C), and LPS, are recognized by TLR2, TLR3, and TLR4, respectively. TLR activation induces the expression of pro-inflammatory mediators, including TNF-α, IFN-β, and iNOS, through MyD88-dependent or TRIF-dependent signaling pathways. Supersulfide donors, such as NAC-S2, suppress the production of these inflammatory mediators by inhibiting the phosphorylation of signaling proteins. NaHS, sodium hydrosulfide.

Simultaneously, the IRAK-TRAF6 complex activates mitogen-activated protein kinase kinase (MKK) cascades, thus leading to activation of activator protein 1 (AP-1), which is another critical transcription factor in inflammation (50). In the TRIF-dependent pathway, TLR4 signaling switches from MyD88 to TRIF after TLR4 internalization into endosomes. TRAM then facilitates the recruitment of TRIF, which triggers expression of IFN-I via the phosphorylation of IFN regulatory factor 3 (IRF3) (**Figure 4**). IFN-I then signals via both autocrine and paracrine processes by binding to the type I interferon receptor (interferon-α/β receptor) (IFNAR). This binding activates the intracellular Janus kinase–signal transducer and activator of transcription (JAK-STAT) pathway, which primarily involves STAT1 and STAT2 (51). Phosphorylated STATs form a complex with IFN regulatory factor 9 (IRF9), which binds to the IFN-I-stimulated response element on the inducible nitric oxide synthase (iNOS) promoter (**Figures 4**, **5**). This complex amplifies iNOS expression alongside other transcription factors (52). Similarly, TLR3 directly engages TRIF or interacts with it via other adaptors, leading to a comparable downstream outcome (53). In addition, other endosomal TLRs, including TLR7-9, can upregulate IFN-I gene expression via IRF7 under certain conditions (38) (**Figure 4**).

### 3.2 RLR-IFN-I signaling pathway

**Figure 6:**
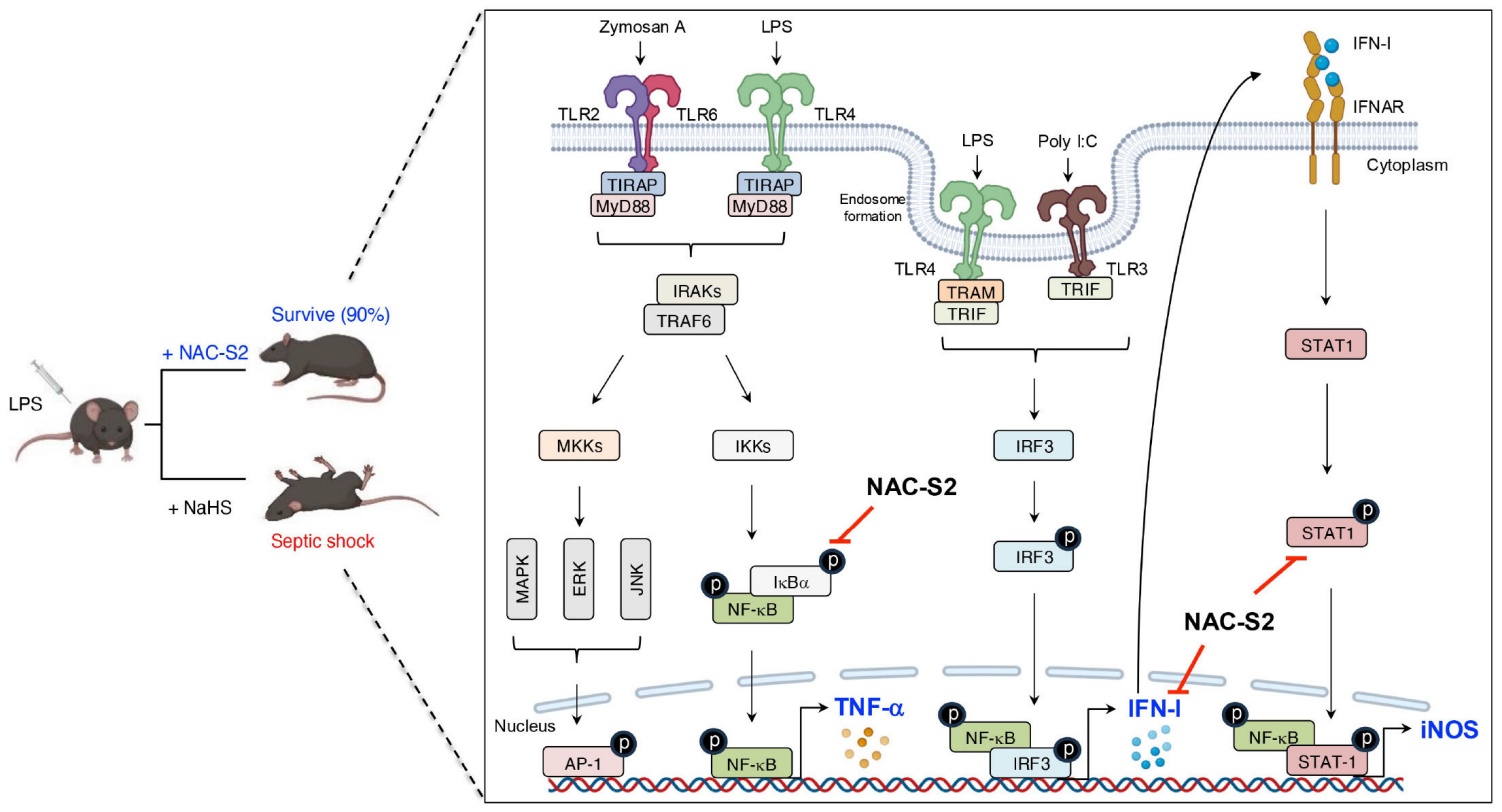
Structural features and signaling pathway of RLRs. The structure and functions of MDA5 are like those of RIG-I. However, MDA5 lacks the repressor domain, which means it does not have self-inhibitory functions. LGP2, however, lacks the CARD domain and therefore cannot transmit signals. The binding of viral RNA to the CTD induces conformational changes in RLRs. These conformational changes facilitate the interaction between MAVS and either RIG-I or MDA5, which leads to the transcription of IFN-I via IRF3-, IRF7-, and NF-κB mediated pathways. LGP2, acts as a modulator and promotes MDA5-mediated signal transduction while suppressing RIG-I-mediated signaling.

**Figure 7:**
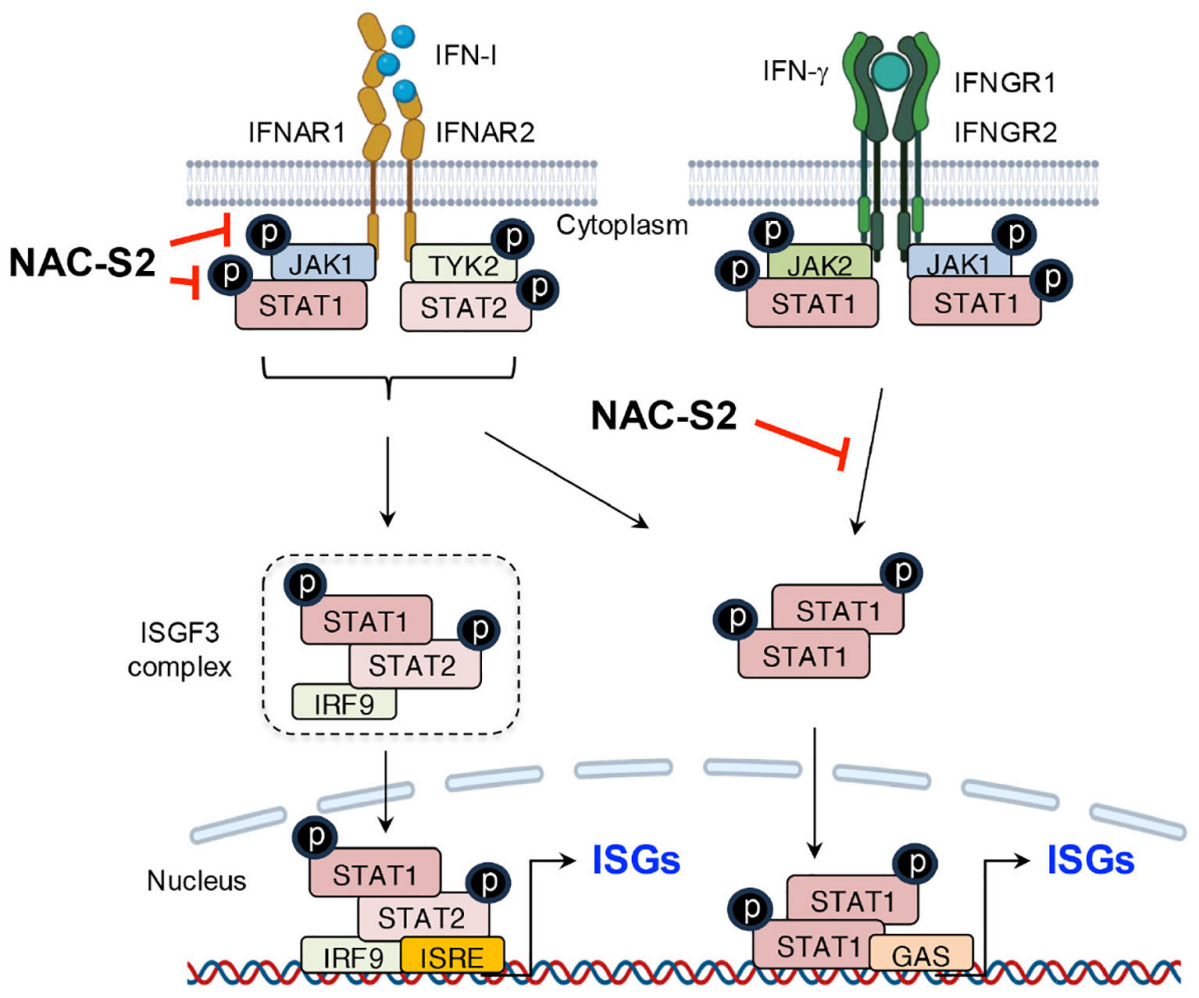
Suppression of IFN signaling by supersulfides. The binding of IFN-I to receptors induces the phosphorylation of JAK1 and TYK2, leading to the release of phosphorylated STAT1/2 heterodimers. These dimers bind to the transcription factor IRF9 and thus form the ISGF3 complex, which translocates to the nucleus and binds to the promoter region of ISRE, thereby activating the transcription of ISGs. Similarly, IFN-γ induces the phosphorylation of STAT1 through its binding to IFN-γ receptors, promoting ISG expression by binding to GAS. NAC-S2 inhibits JAK/STAT signaling by blocking their phosphorylation, thereby reducing ISG expression. TYK2, tyrosine kinase 2; IRF9, IFN regulatory factor 9; ISGF3, IFN-stimulated gene factor 3; ISRE, IFN-stimulated response element; GAS, γ-interferon-activated site; IFNGR1 and IFNGR2, interferon γ receptor 1 and 2.

Other than TLR7 and TLR9, RLRs, including RIG-I, melanoma differentiation-associated gene 5 (MDA5), and laboratory of genetics and physiology 2 (LGP2), serve as intracellular PRRs mainly involved in antiviral immune responses (54). The RIG-I protein is composed of three key parts: two caspase activation and recruitment domain (CARD) at the N-terminus, a repressor domain and the C-terminal domain (CTD) at the C-terminus, and a DexD/H helicase domain in the middle (54). Under normal physiological conditions, RIG-I remains in a self-inhibited state until viral infection. In contrast, MDA5, which shares structural similarities with RIG-I, lacks a repressor domain and therefore does not demonstrate self-inhibitory regulation (54). Unlike RIG-I and MDA5, LGP2 lacks the CARD domain and functions more as a modulator of RIG-I and MDA5 activity. Specifically, LGP2 can negatively regulate RIG-I-mediated recognition of viral dsRNA, thereby reducing the production of IFN-I and other inflammatory mediators and leading to the suppression of antiviral responses (41). Conversely, LGP2 promotes MDA5-mediated antiviral IFN responses in a dose-dependent manner (55). After virus invasion, RIG-I and MDA5 are activated and initiate downstream signaling through CARD-CARD interaction with mitochondrial antiviral signaling protein (MAVS). This interaction triggers the production of IFN-I. As described earlier, secreted IFN-I binds to its receptor, activating the JAK-STAT signaling pathway and inducing the expression of IFN-stimulated genes (ISGs) (56) (**Figures 6**, **7**).

### 3.3 NLRs and inflammasomes

NLRs play crucial roles not only in sensing PAMPs and DAMPs but also in detecting disturbances in cellular homeostasis, such as redox imbalance, ion flux, and other forms of cellular stress (57). During the past two decades, the number of extensive *in silico* analyses increased, and these analyses identified more than 30 NLR family members (57). In structural terms, most NLRs share three key domains: a C-terminal leucine-rich repeat domain, a centrally located nucleotide-binding domain (NACHT), and an N-terminal effector domain. The leucine-rich repeat domain mediates the recognition of PAMPs and DAMPs, whereas the NACHT domain, with its ATPase activity, regulates self-oligomerization. As a noteworthy finding, the effector domain is the most distinctive feature of NLR proteins and serves as the basis for their classification into five subfamilies: NLRA, NLRB, NLRC, NLRP, and NLRX (58). Among these, the NLRP subfamily, which is characterized by the presence of a pyrin domain at the N-terminus, is the largest and most extensively studied group.

**Figure 8:**
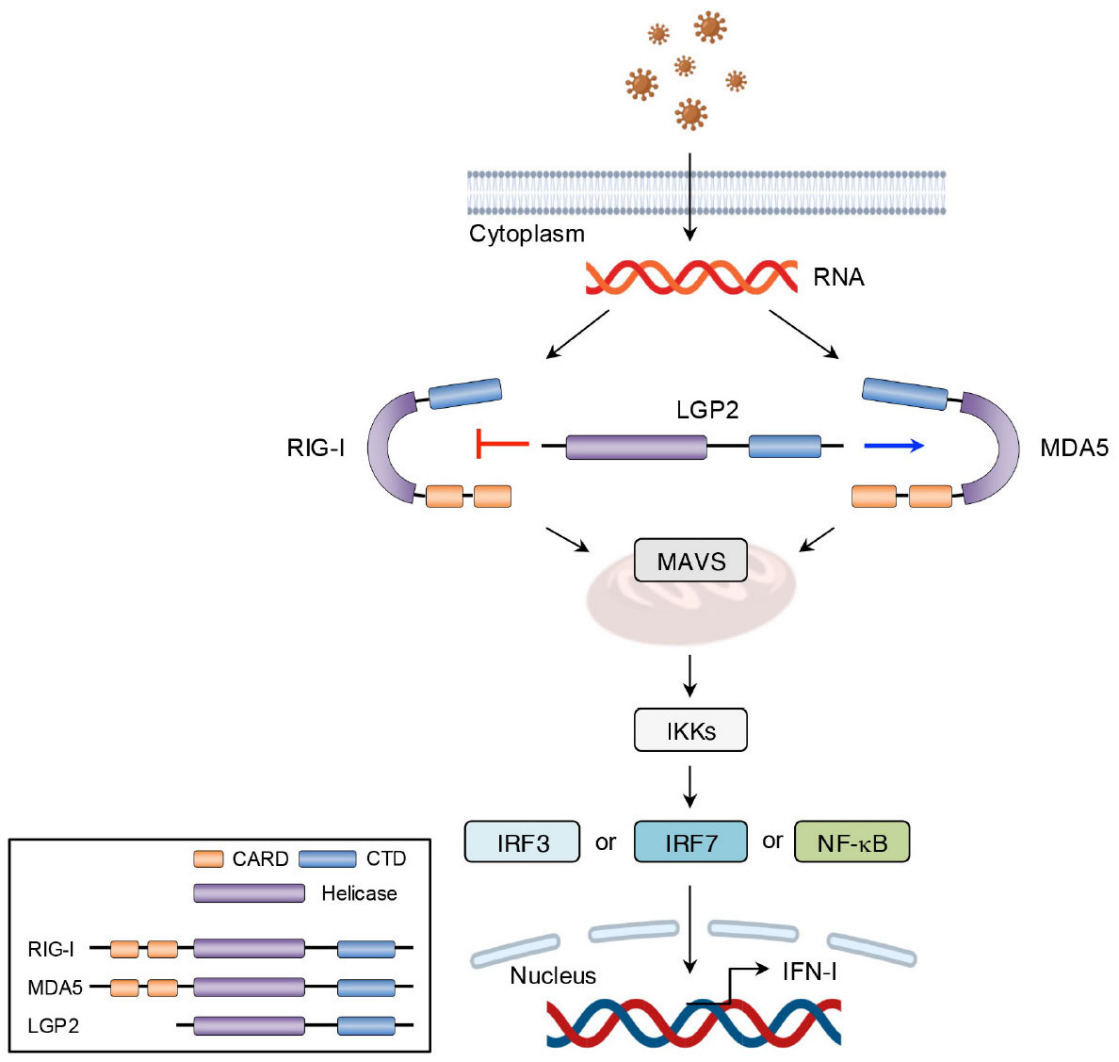
The mechanisms of NLRP3 inflammasome activation. NLRP3 inflammasome activation occurs via two distinct steps. During the priming step, transcriptional upregulation of NLRP3 inflammasome components including NLRP3, pro-caspase-1, and the immature form of cytokines is induced by the recognition of PAPMs or cytokines, leading to NF-κB signaling activation. The activation step is triggered by diverse DAMPs, such as extracellular ATP, pore-forming toxins, ionophore, and crystalline particles. Alterations in cellular homeostasis during this step are believed to represent a common event upstream of NLRP3 inflammasome complex assembly. This perturbation recruits components proteins, resulting in the processing of NLRP3 inflammasome and the maturation of cytokines, including IL-1β and IL-18. IL-1R, interleukin-1 receptor; Nek7, NIMA-related kinase 7.

With regard to function, most NLRPs are involved in immune and inflammatory responses by means of the assembly of a multiprotein platform known as the inflammasome (59). Despite minor variations depend on the specific NLRP involved, inflammasomes generally share a conserved structure comprising three main components: caspase-1, which processes pro-IL-1β and pro-IL-18 into their active forms; a bridging adaptor protein, apoptosis-associated speck-like protein containing a CARD (ASC); and an NLR protein, which serves as the sensor molecule for diverse stimuli (60). A prototypical example is the NLRP3 inflammasome, which is one of the most intensively studied inflammasomes because of its ability to sense a wide array of stimuli (61). After exposure to PAMPs and/or DAMPs, as well as perturbations in cells, NLRP3 expression is upregulated via the NF-κB-mediated signaling pathway. NLRP3 then undergoes dimerization and recruits ASC through homotypic pyrin domain-pyrin domain interactions, thus promoting oligomerization and formation of a larger complex. This assembly subsequently activates caspase-1 through CARD-CARD interactions, thereby completing the NLRP3 inflammasome, which drives immune and inflammatory responses by facilitating the maturation of IL-1β and IL-18 and inducing pyroptosis (62) (**Figure 8**).

## 4 PRR-related inflammatory diseases and current therapies

Inflammatory mediators, such as cytokines, are key regulators of inflammation, and the release of these mediators is typically intended to prevent additional damage. However, excessive or aberrant production of inflammatory mediators caused by dysregulated PRR signaling pathways could contribute to the progression of various diseases (63).

### 4.1 Autoinflammatory diseases

Autoinflammatory diseases comprise a diverse group of disorders driven by innate immune system dysregulation (64). This dysregulation is frequently linked to gene mutations, which either impair the function of anti-inflammatory genes or enhance pro-inflammatory gene activity. Such genetic alterations result in unregulated inflammatory responses and excessive production of cytokines such as IL-1β, TNF-α, and IFN-I. These cytokine-associated pathways underlie the inflammasomopathies, relopathies, and interferonopathies, respectively (65). Advances in genomics have significantly increased the identification of novel autoinflammatory conditions, with the Infever database (https://infevers.umai-montpellier.fr/web/) cataloging more than 50 monogenic disorders to date, with additional categories continually emerging (66, 67) (**Table 1**).

**Table 1:** Typical monogenetic autoinflammatory diseases.

Subtype	Name	Gene	Protein	Phenotype features
Inflammasomopathies	FMF	*MEFV (NM_000243.2)*	Pyrin	Fever (12-72 hours), serositis, non-erosive acute arthritis of large joints, erysipelas-like lower extremity rash
	PAAND	*MEFV (S242R)*	Pyrin	Fever, neutrophilic dermatosis, acne, pyoderma gangrenosum, cutaneous abscesses
	CAPS	*NLRP3 (NM_004895.4)*	Cryopyrin/NLRP3	FCAS: cold-triggered episodes of fever, urticaria, conjunctivitis; MWS: cold-induced urticaria, sensorineural hearing loss; CINCA: neonatal onset, urticaria, chronic aseptic meningitis, deforming arthropathy, facial dysmorphia
	FCAS2	*NLRP12 (NM_144687.3)*	Monarch 1/NLRP12	Fever, cold-induced urticaria urticaria, arthralgia
	FCAS4	*NLRC4 (NM_001199138.2)*	NLRC4	Neonatal onset, cold-induced urticaria, arthralgia, fever
	AIFEC	*NLRC4 (NM_001199138.2)*	NLRC4	Early-onset enterocolitis, recurrent MAS
Relopathies	TRAPS	*TNFRSF1A (NM_001065.4)*	TNF receptor superfamily member 1A	Fever (>7–14 days), periorbital edema, conjunctivitis, pseudo-cellulitis rash, abdominal pain, migrating myalgia, arthralgia, chest pain, lymphadenopathy
	HA20	*TNFAIP3 (NM_001270508.2)*	A20	Fever, oral, gastrointestinal and genital ulcerations, arthritis, uveitis (mimic Behçet’s disease)
	LUBAC deficiency	*HOIL1 (NM_031229.4)* *HOIP (NM_017999.5)*	HOIL1 HOIP	Fever, immunodeficiency, hepatosplenomegaly, amylopectin-like deposits in muscles
Type I interferonopathies	AGS	*MDA5 (NM_022168.4)*	Melanoma differentiation-associated protein 5	Fever, neurologic decline, chilblains, and IgA deficiency
	SAVI	*STING (NM_198282.4)*	Stimulator of interferon gene	Skin vasculopathy, bilateral interstitial lung disease
	PRAAS	*PSMA3/PSMB8, PSMB4/PSMB9, PSMB4/PSMB8, PSMB9 (GRCh38:6:32,854,191-32,859,850), PSMB10 (GRCh38:16:67,934,505-67,936,849), PSMB7 (GRCh38:9:124,353,464-124,415,441), PSMA3 (GRCh38:14:58,244,842-58,272,003), POMP (GRCh38:13:28,659,129-28,678,958), PSMG2 (GRCh38:18:12,658,737-12,725,739)*	Proteasome complex subunit and proteosome chaperone	Fever, joint contractures, annular plaques, eyelid swelling, hepatosplenomegaly, developmental delay, and anemia
	ISG15 deficiency	*ISG15 (NM_005101.4)*	Interferon-stimulated gene 15	Neurological involvement, mycobacterial susceptibility

FMF, familial Mediterranean fever; PAAND, pyrin-associated auto-inflammation with neutrophilic dermatosis; CAPS, cryopyrin-associated periodic syndromes; FCAS, familial cold autoinflammatory syndrome; AIFEC, autoinflammation and infantile enterocolitis; TRAPS, TNF receptor-associated periodic syndrome; HA20, A20 haploinsufficiency; LUBAC, linear ubiquitin chain assembly complex; AGS, Aicardi-Goutières syndrome; interferon induced with helicase C domain 1; STING, stimulator of interferon genes; SAVI, STING-associated vasculopathy with onset in infancy; PRAAS, proteasome-associated autoinflammatory syndromes; ISG, interferon-stimulated gene; MWS, Muckle-Wells syndrome; CINCA, chronic infantile neurologic cutaneous articular syndrome; MAS, macrophage activation syndrome.

One prominent example of these disorders is the cryopyrin-associated periodic syndrome (CAPS), which comprise a spectrum of disorders caused by gain-of-function mutations in the NLRP3 gene (also referred to cold-induced autoinflammatory syndrome 1, or *CIAS1* gene) on chromosome 1q44, which encodes cryopyrin. These mutations lead to hyperactivation of the NLRP3 inflammasome and persistent overproduction of IL-1β (68, 69). Categories of CAPS include three clinical subtypes: familial cold autoinflammatory syndrome (FCAS), Muckle-Wells syndrome (MWS), and chronic infantile neurologic cutaneous articular (CINCA) syndrome, also known as neonatal-onset multisystem inflammatory disease (NOMID). FCAS is characterized by episodic fever, rash, conjunctivitis, and arthralgia triggered by cold exposure. MWS presents with chronic urticaria, sensorineural hearing loss, and an elevated risk of AA amyloidosis in approximately 25% of cases. CINCA/NOMID manifests early in life with a non-pruritic rash, aseptic meningitis, deforming arthropathy, skeletal abnormalities, and distinctive facial features such as frontal bossing and saddle-nose deformity (70). Over 200 variants of the CIAS1 gene, predominantly in exon 3, have now been identified and show strong genotype-phenotype correlations (67). However, genetic analysis may not always detect pathogenic mutations that are due to mosaicism or unidentified epigenetic factors (71). Additionally, CAPS-like symptoms may arise from mutations in other related genes, such as *NLRP12* and *NLRC4* (68).

### 4.2 Systemic inflammatory response syndrome and sepsis

Since the onset of the coronavirus disease 2019 (COVID-19) pandemic, SIRS has gained attention because of its association with cytokine storm management efforts aimed at reducing morbidity and mortality (72). SIRS is an exaggerated immune response to harmful stimuli and is intended to contain and eliminate the source of injury. However, excessive cytokine release can lead to a massive inflammatory cascade, which causes reversible or irreversible organ dysfunction and even death (73). SIRS associated with a suspected infection is classified as sepsis, which may progress to septic shock, with increasing mortality along this continuum (74). Both conditions reflect a progressively worsening imbalance between pro- and anti-inflammatory responses within the body.

Recent studies in sepsis models and patients have demonstrated markedly elevated NF-κB activity across multiple organs. Higher NF-κB activity correlates with increased mortality and poorer clinical outcomes, underscoring the critical role of NF-κB signaling in SIRS pathology (75). Various TLRs detect PAMPs, such as lipoproteins recognized by TLR1/2, LPS and β-glucans by TLR4, and bacterial flagellin by TLR5 (76–78). These interactions activate NF-κB through distinct signaling pathways. For instance, LPS-induced TLR4 signaling involves the recruitment of TIRAP and MyD88, followed by activation of IRAK1 and IRAK4 with the assistance of TRAF6 (79). This IRAK/TRAF6 complex dissociates from TLR4, interacts with TGF-β-activating kinase-1 (TAK1) and its associated TAK-binding proteins, and thus forms the TRAF6/TAB/TAK1 complex (80). Subsequently, degradation of IRAK1 leads to the activation of IKKα and IKKβ, which, in turn, triggers NF-κB activation and downstream mediator production (79). Notably, LPS-induced NF-κB activation occurs in two phases: an early phase (0.5-2 hours after stimulation) driven by TLR4 signaling, and a late phase (8-12 hours after stimulation) sustained by cytokines such like TNF-α and IL-1β generated during the early phase (81). This biphasic activation likely contributes to the progression from sepsis to septic shock.

### 4.3 Cancer

Chronic inflammation, apart from the genetic and systemic inflammation, is critically implicated in cancer development (82, 83). Tumors are initiated when normal cells acquire mutations that confer growth and survival advantages (84). A well-established example is *Helicobacter pylori*-induced gastric cancer, in which bone marrow-derived cells are recruited to the injury site. These cells exhibit greater plasticity and are predisposed to progressing through the metaplasia-dysplasia-cancer sequence (85). Recent findings indicate that chronic inflammation fosters genetic instability, increasing the likelihood of genetic alterations (84). Furthermore, studies of *H. pylori*-induced gastritis have revealed a strong correlation between gastric cancer risk and DNA methylation (86).

The inflammatory tumor microenvironment is now recognized as a significant contributor to tumor growth and progression (87). PRRs are highly expressed in various tumor tissues, including colon, lung, breast and gastric cancer, plus melanoma (88). Activation of PRRs in tumor cells triggers the release of cytokines, which is a key factor in establishing a pro-tumor inflammatory microenvironment (89). This microenvironment is rich in immune cells, particularly tumor-associated macrophages (TAMs) and T lymphocytes (90). Depending on their subtype, TAMs can produce either pro-inflammatory cytokines (e.g., IL-1β and IL-6) or anti-inflammatory cytokines (e.g., IL-4 and IL-10), thereby modulating the cytokine profile and shifting the tumor microenvironment toward either tumor suppression or tumor promotion (91). In addition, tumor cells secrete vascular endothelial growth factor (VEGF) to recruit TAMs, which become a major source of angiogenic factors via NF-κB and STAT3 activation (92). T cells also exhibit dual roles in a tumor microenvironment via cytokines secretion (93). However, malignancy often suppresses an effective T cell response, and regulatory T cells are frequently recruited to tumors, thereby continuing to dampen anti-tumor immunity (94, 95).

### 4.4 Limitations of current anti-inflammatory therapies

For decades, nonsteroidal anti-inflammatory drugs (NSAIDs) such as aspirin and ibuprofen have been the cornerstone of managing inflammatory symptoms (96). Molecularly, these drugs work by inhibiting cyclooxygenases and reducing prostaglandin E2 production, which is a key mediator of inflammation (97). However, this mechanism also underlies significant adverse effects, including gastrointestinal complications such as ulcers and bleeding, as well as cardiovascular risks associated with cyclooxygenase-selective inhibitors (97, 98). More recently, therapies that target inflammatory cytokines have gained attraction. These agents act as antagonists that neutralize specific cytokines or block their receptors, thereby curbing inflammation (99). Clinical studies demonstrated their marked efficacy in managing various inflammatory diseases (100, 101). Nevertheless, systemic cytokine inhibition poses risks, primarily by weakening host defenses, which is a considerable limitation (102). Therefore, tailoring new therapies to target multiple inflammatory signaling pathways could lead to more effective anti-inflammatory treatments while minimizing the risk of severe side effects.

## 5 Versatile therapeutic potentials of supersulfide donors in inflammatory diseases

**Figure 9:**
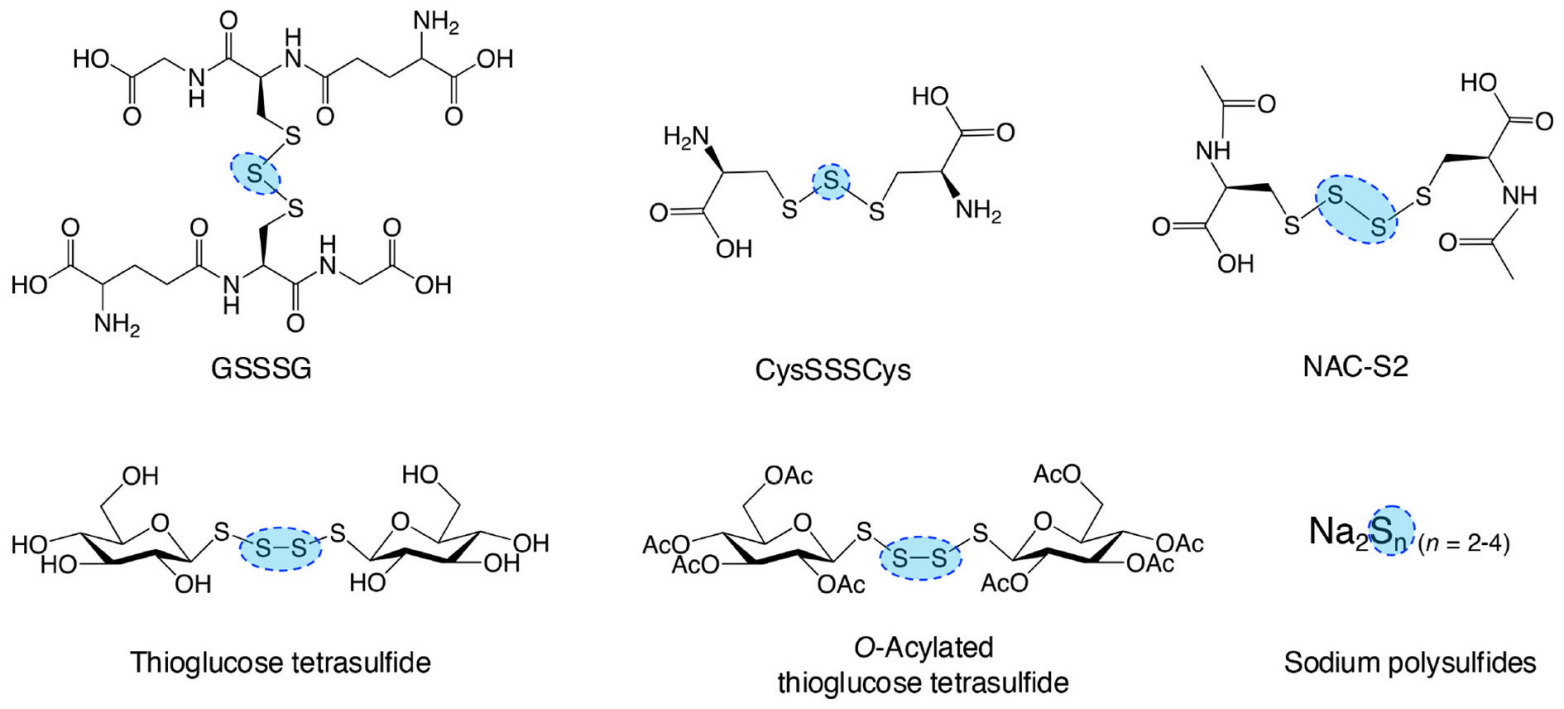
Typical supersulfide donors. The diagram illustrates various synthetic supersulfide donors, including both organic and inorganic compounds, some of which are endogenously produced. These compounds serve as valuable tools for investigating the biological functions of supersulfides and their therapeutic potential both *in vitro* and *in vivo* studies.

Endogenous and synthetic supersulfide donors, as summarized in **Figure 9**, can penetrate cells and organs, delivering sulfur atoms to intracellular acceptors, thereby elevating supersulfide levels (103, 104). This section highlights the therapeutic potential of supersulfides in managing inflammatory diseases, as supported by evidence from studies utilizing supersulfide donor treatments.

### 5.1 Prevention of septic shock in mice by suppressing NF-κB signaling pathway

In the mouse macrophage cell line RAW264.7 cells, stimulation with LPS induced early-phase production of TNF-α. Analysis of these cells revealed phosphorylation of both IκBα and NF-κB, which indicates activation of the TLR4 signaling pathway. Under these conditions, treatment with the synthetic supersulfide donor *N*-acetylcysteine tetrasulfide (NAC-S2) significantly elevated intracellular levels of LWM supersulfides. Interestingly, LPS-induced phosphorylation of both IκBα and NF-κB was strongly inhibited in cells treated with NAC-S2, thereby suppressing downstream cytokine production of TNF-α. In contrast, NAC-S2 treatment did not affect adjacent signaling pathways, as evidenced by irregular phosphorylation of mitogen-activated protein kinase (MAPK), c-Jun N-terminal kinase (JNK), and extracellular signal-regulated kinase (ERK). This result suggests that supersulfides specifically target the NF-κB signaling pathway (105). In addition to TLR4, NAC-S2 potently inhibited other TLR-mediated NF-κB signaling pathways, as shown by the suppression of TNF-α production in macrophages stimulated with ligands for TLR2 and TLR3 in the presence of NAC-S2 (105). Similarly, the endogenous supersulfide donor GSSSG inhibited the NF-κB signaling pathway by suppressing LPS-induced inflammatory profiling in both mouse and human epithelial cells (106).

In a mouse model of septic shock, administration of LPS reduced the survival rate to 20%. As a notable finding, treatment with NAC-S2 improved the survival rate to 90% and significantly ameliorated inflammatory responses (105). In contrast, no improvement in survival rate was observed in mice treated with H_2_S donor sodium hydrogen sulfide, indicating that the anti-inflammatory effects of NAC-S2 are far superior to those of H_2_S donor (**Figure 5**).

### 5.2 Inhibition of IFN signaling by blocking the JAK/STAT signaling pathway

As mentioned above, persistent activation of TLR4 leads to IFN-β production, which subsequently induces iNOS expression through the STAT signaling pathway during the late phase of inflammation (52, 105). This pathway was also investigated in RAW264.7 cells, where IFN-β production, accompanied by STAT-1 phosphorylation, was detected 6 hours after LPS exposure. iNOS expression was abolished when RAW264.7 cells were treated together with either NAC-S2 or thioglucose tetrasulfide, another synthetic supersulfide donor (107, 108) (**Figure 5**).

Upstream analysis revealed that NAC-S2 treatment blocked STAT-1 phosphorylation by suppressing IFN-β production induced by sustained LPS exposure (105, 108). Further investigation showed that exposure of RAW264.7 cells to recombinant IFN-I, including IFN-α and IFN-β, activated the STAT-1 signaling pathway via phosphorylation of JAK1 and tyrosine kinase 2 (TYK2). Treatment with either NAC-S2 or thioglucose tetrasulfide significantly suppressed iNOS expression in the presence of IFN-I, indicating that supersulfides effectively inhibit IFN-I signaling. These inhibitory effects were attributed to the negative modulation of JAK1 phosphorylation, whereas TYK2 phosphorylation remained undetected (108). Also noteworthy is that NAC-S2 inhibited not only INF-I- but also IFN-γ-induced signaling, which highlighting the broader immunomodulatory potential of supersulfides (**Figure 7**).

### 5.3 Suppression of IL-1β production by modulating the NLRP3 inflammasome

One study revealed a negative correlation between CARS2 expression and the production of inflammatory cytokines, such as IL-1β (109). In macrophages deficient in the cystine/glutamate antiporter xCT, CysSSH levels were significantly reduced compared with those in wild-type macrophages, probably because of an insufficient availability of CysSH as a substrate. Notably, xCT-deficient macrophages exhibited an upregulation of pro-inflammatory genes, including IL-1β and TNF-α. Upon exposure of these macrophages to LPS, the enhancement of intracellular supersulfides via treatment with NAC-S2 almost completely suppressed IL-1β production, which suggests that supersulfides establish a negative feedback loop regulating NLRP3 inflammasome-mediated IL-1β production (110).

**Figure 10:**
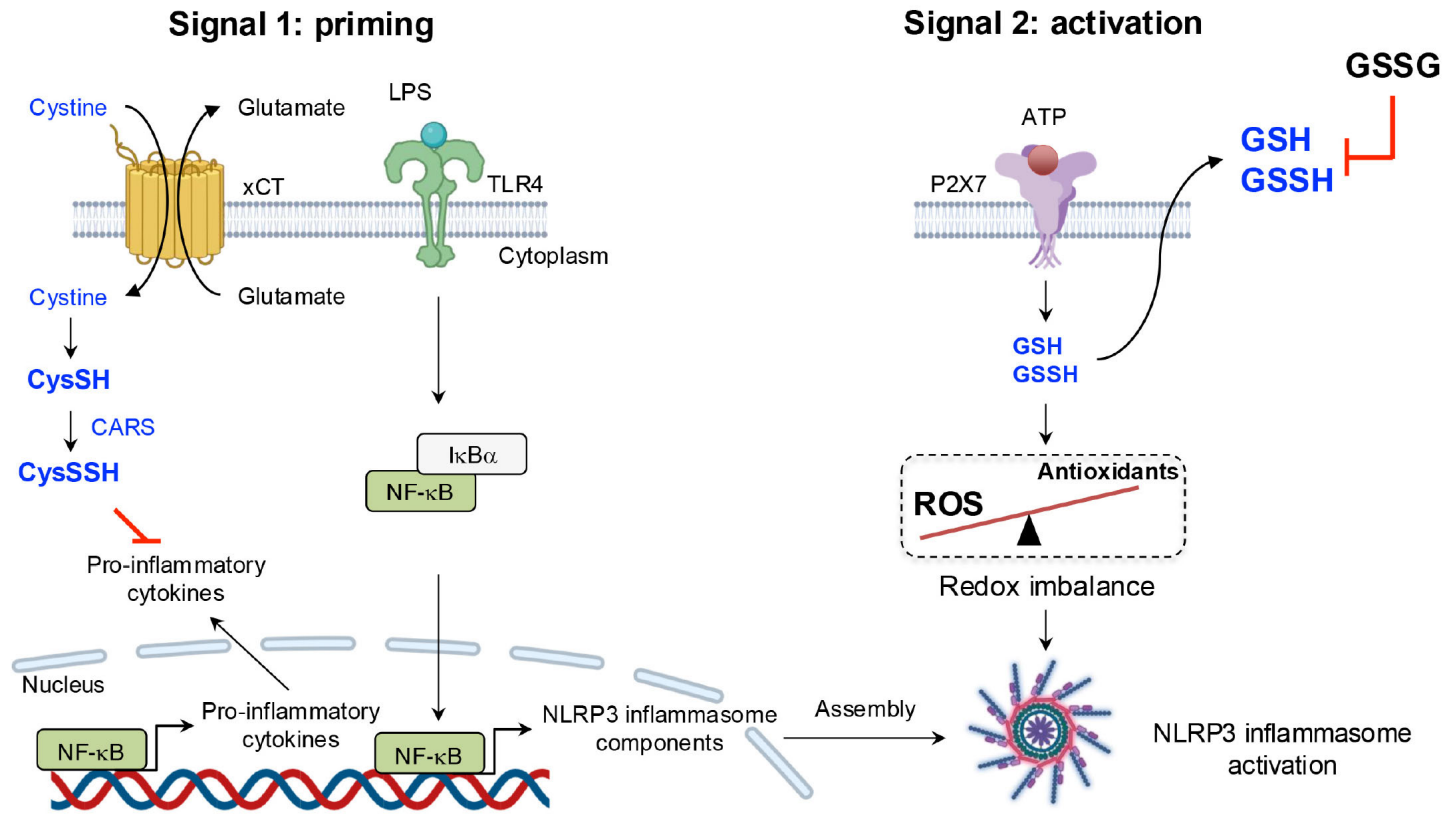
Negative regulation of the NLRP3 inflammasome by supersulfides. LPS priming induces the upregulation of *slc7a11*, which encodes the cystine transporter xCT. Increased xCT expression enhances cystine uptake, promoting the production of CysSSH via CARS and establishing a negative feedback loop to limit excessive inflammatory mediator production. ATP sensing by the P2X purinoceptor 7 (P2X7) receptor, however, triggers the efflux of GSH and GSSH, along with ROS accumulation, leading to redox imbalance. This imbalance activates the NLRP3 inflammasome. Suppression of GSSH efflux through exogenous GSSG administration significantly inhibits NLRP3 inflammasome activation, underscoring the regulatory role of supersulfides in inflammation.

Although the precise molecular mechanisms of the NLRP3 inflammasome activation remain to be fully elucidated, reactive oxygen species (ROS) are widely regarded as essential for NLRP3 inflammasome activation (111). Zhang et al. demonstrated that GSSH efflux occurs proximal upstream of NLRP3 inflammasome activation after exposure to DAMPs, the result being ROS accumulation and redox imbalance, which collectively trigger inflammasome activation. An important finding was that suppression of GSSH efflux through the extracellular addition of GSH significantly inhibited NLRP3 inflammasome activation and subsequent IL-1β release (112). Furthermore, a recent study emphasized that supplementation with CysSSH protected macrophages from DAMPs-induced pyroptosis by modulating NLRP3 persulfidation, providing valuable insights into the regulatory role of supersulfides in inflammasome activation (113) (**Figure 10**).

### 5.4 Protective effects on pulmonary disease via multifaceted mechanisms

Respiratory infectious diseases remain a significant global challenge, as demonstrated by the COVID-19 pandemic, which was caused by severe acute respiratory syndrome coronavirus 2 (SARS-CoV-2) and annual influenza outbreaks (114). The clinical spectrum of COVID-19 ranges from mild respiratory symptoms to severe disease manifestations, including pneumonia, acute respiratory failure, multiorgan failure, and death (115). Increasing evidence links the molecular pathology of COVID-19 to a cytokine storm, characterized by the excessive and uncontrolled release of pro-inflammatory cytokines, such as IL-1β, TNF-α and IL-6 (116, 117). These cytokines then stimulate the generation of ROS by means of enzymatic reactions or damaged mitochondria (118). ROS, such as superoxide and hydrogen peroxide, amplify inflammatory responses and oxidative stress, thereby causing secondary tissue damage (119). However, current COVID-19 therapies are limited, as they often target either free radicals or inflammatory responses in isolation rather than addressing both simultaneously (120).

**Figure 11:**
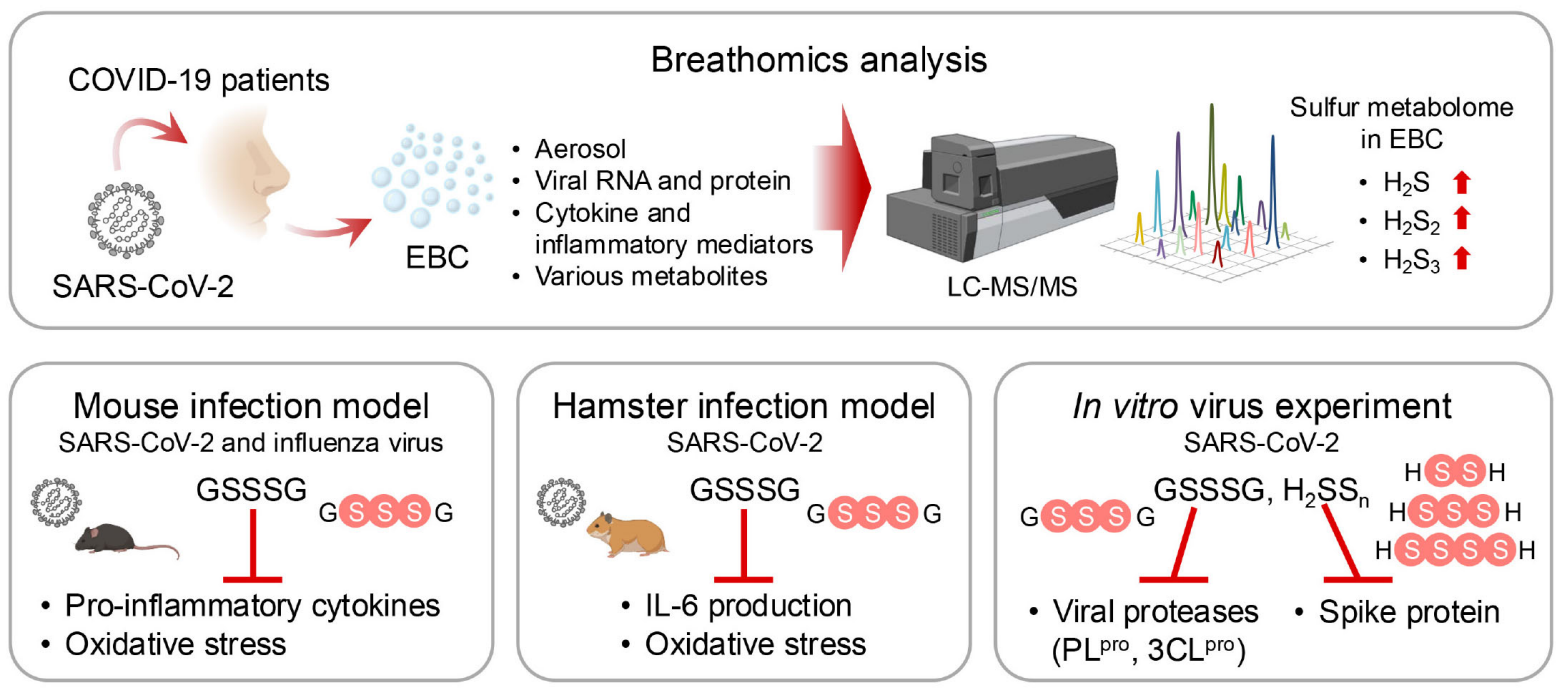
Protective effects of supersulfides in pulmonary disease. Liquid chromatography-mass spectrometry (LC-MS/MS)-based breathomics analyses have identified supersulfides in EBCs from COVID-19 patients. Supersulfides manifest protective effects against viral infections by using three key mechanisms: suppression of pro-inflammatory cytokine production, inhibition of oxidative stress, and modification of viral proteases. These effects were validated by treatments with supersulfide donors in various animal models.

The role of supersulfides in viral infections has garnered increasing attention (16). Studies showed that in exhaled breath condensate (EBC) from patients infected with SARS-CoV-2, the amounts of supersulfide metabolites, such as H_2_S_1-3_ and thiosulfate, are significantly elevated compared with those in healthy individuals. Sulfur-omics analyses indicated that the levels of these metabolites increase with disease progression and that the metabolites are excreted through EBCs. In mouse infection models of both SARS-CoV-2 and influenza A, infection induced significant production of pro-inflammatory cytokines and oxidative stress. Treatment with GSSSG or inorganic supersulfide donors strongly inhibited IL-6 production and alleviated oxidative stress. Also, supersulfide donors blocked viral entry into host cells by directly inactivating viral spike proteins and inhibited viral replication by targeting key proteases, such as the papain-like protease and 3CL protease in SARS-CoV-2. These findings highlight the protective effects of supersulfides against viral infections through multiple mechanisms (**Figure 11**).

In addition to viral infections, chronic obstructive pulmonary disease (COPD) is believed to be another global health crisis and is the fourth leading cause of death worldwide (121). COPD is characterized by progressive airflow limitation due to small airway remodeling and lung parenchyma destruction from emphysema, driven by chronic inflammation and oxidative stress (122). While direct therapeutic evidence is lacking, clinical studies suggest a negative correlation between supersulfide levels and COPD in patients with both smoking-induced COPD and asthma-COPD overlap syndromes (123, 124). For instance, CARS2 expression was significantly lower in patients with COPD than in healthy donors (16). Moreover, CARS2 heterozygous knockout mice, when exposed to elastase or cigarette smoke extract to induce COPD, manifested more severe disease phenotypes than did wild-type mice, thereby underscoring the protective role of supersulfides in COPD (16).

## 6 Conclusion and future perspectives

This review highlights the chemical and biological properties of supersulfides and emphasizes the dynamic changes of supersulfides during various inflammatory diseases. Evidence strongly supports the significant therapeutic potential of supersulfides in modulating inflammation, with supersulfide donors emerging as a promising strategy for therapeutic intervention. However, despite this potential, several challenges hinder the clinical application of supersulfides. Key gaps remain in understanding the mechanisms governing supersulfide metabolism and their interactions of supersulfides with proteins. Also, issues related to the stability, bioavailability, and pharmacokinetics of supersulfide donors must be addressed to optimize their therapeutic efficacy and minimize potential side effects. By overcoming these challenges, the full therapeutic potential of supersulfides can be realized, which may lead to innovative and effective treatments for uncontrolled inflammatory conditions. Computational approaches, including density functional theory (DFT), absorption, distribution, metabolism, excretion, and toxicity (ADMET) analysis, and molecular docking, have been employed to investigate the structural and biological properties of molecule (125, 126). Future studies should also integrate these methods to elucidate the molecular mechanisms of supersulfide interactions and facilitate rational drug design. By combining experimental and computational approaches, deeper insights into supersulfide reactivity and their biological targets can be achieved, further accelerating drug development efforts.
